# The influence of prior laparoscopic experience on learning laparoendoscopic single site surgery: a prospective comparative preliminary study using cystorraphy in a live porcine model

**DOI:** 10.1186/s12894-017-0242-2

**Published:** 2017-07-12

**Authors:** U-Syn Ha, Kyu Won Lee, Sun Wook Kim, Seung Hyun Jeon, Tae Gyun Kwon, Hyung Keun Park, Sung-Hoo Hong

**Affiliations:** 1 Department of Urology, Seoul St. Mary’s Hospital, College of Medicine, The Catholic University of Korea, 222, Banpo-daero, Seocho-gu, Seoul, 06591 Republic of Korea; 2 Department of Urology, School of Medicine, Kyung Hee University, Seoul, Republic of Korea; 3 Department of Urology, College of Medicine, Kyungpook National University, Daegu, Republic of Korea; 4 Department of Urology, Asan Medical Center, University of Ulsan College of Medicine, Seoul, Republic of Korea

**Keywords:** Laparoscopic surgery, Learning curve, Minimally invasive surgery

## Abstract

**Background:**

The purpose of this study is to assess the impact of prior laparoscopic experience on the ability to learn laparoendoscopic single site surgery (LESS) skills.

**Methods:**

A total of 33 urologists who completed a training program in LESS surgery were recruited for this study. After completing the educational course and training, the study participants demonstrated LESS suturing and knot-tying via a 2-cm cystotomy in a live porcine model for 15 min. An objective structured assessment of technical skills (OSATS) was used to evaluate videos of each participant’s procedure. The participants were divided according to laparoscopic experience; advanced experienced group (AS), intermediate experienced group (IS), novice group (NS).

**Results:**

Three participants in the NS group completed the porcine cystorrhaphy in 15 min (30.0%), 3 (25.0%) completed the task in the IS group, and 3 (27.2%) completed it in the AS group. There were no statistically significant differences in the mean total OSATS quality score (NS; 16.7, IS; 18.5, AS; 16.8) among the 3 groups. Concerning all each assessment, there were also no statistically significant difference. Additionaly, the mean total OSATS quantity score (NS; 4.1, IS; 3.5, AS; 4.3) did not differ significantly among groups. The NS group succeeded a mean of 1.4 knots, the IS group succeeded 0.9, and the AS group 1.3 (*p* = 0.727).

**Conclusions:**

There was no significant difference among the groups in LESS proficiency after training. Surgeons who were novices in conventional laparoscopic surgery reached comparable scores to those of experienced laparoscopic surgeons after training.

## Background

Minimally invasive surgeries are becoming more widely accepted and popular procedures. Advanced surgical techniques and new instruments allow surgeons to minimize surgery’s invasiveness. Most recently, laparoendoscopic single-site surgery (LESS) has become an effective surgical alternative to conventional laparoscopic surgery [1, 2].

The surgical techniques required to perform LESS are different from those required during conventional laparoscopic surgery [3]. LESS is technically challenging because the surgeon must pass all of his instruments through a single port to provide limited triangulation [4]. To overcome this challenge, surgeons can operate either using one instrument, or using articulating instruments that cross over each other [5]. These limitations increase the challenge of learning LESS, which requires considerable practice.

The LESS learning curve can be influenced by several factors, including technical advances, education and prior surgical experience. Simulation is a common learning tool used to teach surgeons the basic technical skills of conventional laparoscopy [6]. It allows surgeons to acquire skills for laparoscopic surgery without the risk of harming patients. It is expected that simulation could also be a valuable learning tool for LESS. We investigated how a surgeon’s prior experience influences the LESS learning curve.

Proficiency in LESS likely requires experience with minimally invasive surgery, including laparoscopic surgery. Therefore, experience in laparoscopic surgery could shorten the LESS learning curve. However, several prior studies have found conflicting results. Lewis et al. [7] demonstrated that previous laparoscopic experience improved the ability to perform LESS. In contrast, a comparative study evaluating the LESS learning curves of novices by Sodergren et al. [8] found that prior training in conventional laparoscopy does not influence LESS learning or proficiency.

However, the Sodergren et al. study has limitations. For example, the tasks used as assessments are basic laparoscopic skills that may not have provided a sufficient challenge to experienced laparoscopic surgeons. Therefore, more complex tasks may have exposed differences. In addition, this study did not provide proper time for learning, because the tasks were tested on a single attempt, without prior training or education (3). The group also used a box trainer, with which they could not assess performance under biological conditions. In addition, the study is so insufficient not to draw conclusion in view of the results so far achieved. Given the limitations of the prior research in this area, our aim was to conduct an optimized study, and to provide more satisfactory results.

Surgeons who participated in this study had completed a training program and were assessed using difficult tasks, such as cystorrhaphy. The purpose of this study was to assess whether prior laparoscopic surgical experience affects the ability to learn LESS skills.

## Methods

### Subjects

The study was approved by the Institutional Animal Care and Use Committee of Samsung Biomedical Research Institute (H-B2–001). The animals for this study were used for LESS training courses, in which the bladder had been preserved intact.

Of 36 urology specialists who received surgical training program in the LESS workshop, 33 were suitable for this study, and were recruited. The suitable participants included novices with LESS experience and surgeons with varying laparoscopic experience. Before survey, written informed consent had been taken from all participants. We surveyed participants for prior laparoscopic surgical experience during registration for the workshop (Figure 3 in Appendix). In order to be included, participants must have completed the training program and taken the test.

**Fig. 1:**
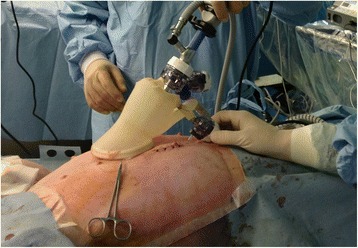
External view of Laparoendoscopic single site surgery homemade single-port device using a surgical glove, wound retractor (Alexis, Applied Medical), and trocars

### Training

The workshop was composed of 3 parts under the supervision of tutors. The first part involved theoretical instruction, while the second part included training in basic surgical skills including instrument handling, cutting, grasping, clipping and suturing in the live porcine model and box trainer. During the third part of the workshop, the participants practiced their surgical skills by performing nephrectomy, partial nephrectomy, pyeloplasty, and ureter anastomosis in live porcine models. Each participant had the opportunity to practice his/her respective training format for at least 90 min using the porcine model. All procedures were performed through a single port device that was made from a size 7 surgical glove and an Alexis wound retractor (Applied Medical, Rancho Santa Margarita, CA). Three trocars were inserted through the glove finger site (Fig. 1). The participants were supplied with standard and roticulating dissectors, scissors, and needle holders.

**Fig. 2:**
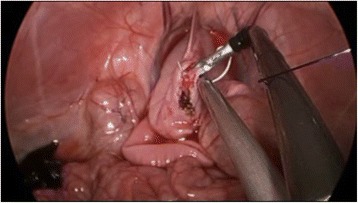
The view of of Laparoendoscopic single site cystorraphy in a live porcine model

### Performance assessment

After finishing the educational course and training, the study participants performed LESS suturing and knot tying via a 2-cm cystotomy in a live porcine model (Fig. 2). Cystorrhaphy was performed for 15 min using closed 2–0 polyglactin sutures. Each participant was recorded while performing cystorrhaphy. Participant videos were blinded with an identifying code. Recorded videos were conveyed to the assessor to be selected for objective structured assessment of technical skills (OSATS).

Two assessors with experience in laparoscopic surgery and LESS scored the cystorrhaphy performances. The OSATS evaluation was used to assess the videos for quantity (time to close the cystostomy, number of knots required, and each step of procedure) and quality (see Appendix). These methods were adopted and edited from Martin et al. [9]. We used the same OSATS that McDougall et al. [10] used to score porcine laparoscopic cystorrhaphy. The participants were assessed on task success, and on whether the closure was watertight or not. The closure’s watertight seal was assessed at the end of the procedure by instilling 150 cm^3^ of saline into the bladder under laparoscopic visualization. A successful cystorrhaphy was one that was completed in 15 min and produced bladder closure with no saline leakage.

### Statistical analysis

The participants were divided into 3 groups according to their prior laparoscopic experience: the novice surgeon group (NS), the intermediate experienced surgeon group (IS), and the advanced experienced surgeon group (AS). Participants with no prior experience as primary operators were placed in the NS group. Primary surgeon with experience in laparoscopic surgery including nephrectomy and cyst marsupialization, but not in reconstructive surgery were placed in the IS group. Finally, primary surgeons with experience in 30 or more cases of laparoscopic reconstructive surgery, including partial nephrectomy, pyeloplasty, and prostatectomy, were placed in the AS group. Data were analyzed statistically and expressed as means ± standard deviations. The scores of each group were compared using ANOVA. Tukey’s test was used for multiple comparisons. Pearson’s chi-square test was used to compare the 3 groups with regard to their success. *P*-values <0.05 were considered statistically significant. Since this was a pilot study, power analysis was not performed.

## Results

Thirty-six urologists participated in workshop. Of these, 33 participants who completed the suture task survey were enrolled. Ten participants were placed in the NS group, 12 in the IS group, and 11 in the AS group. Some participants in group AS had performed over 100 laparoscopic partial nephrectomies. Three participants in the NS group completed the porcine cystorrhaphy in 15 min (30.0%), 3 (25.0%) completed the task in the IS group, and 3 (27.2%) completed it in the AS group with no significant difference. Table 1 shows the mean and total OSATS quality scores from each assessment. There were no statistically significant differences in the mean total OSATS quality score among the 3 groups. Concerning all each assessment, there were also no statistically significant difference. The results of quantity assessment from each group are summarized in Table 2. There was also no statistically significant difference in the mean total OSATS quantity score among the groups. The NS group succeeded a mean of 1.4 knots, the IS group succeeded 0.9, and the AS group 1.3 (*p* = 0.727).

**Table 1:** Results of OSATS quality on each variable and total score

	Novice (*n* = 10)	Intermediate (*n* = 12)	Advanced (*n* = 11)	*p-*value
Respect	2.8 ± 1.3	3.4 ± 1.2	2.8 ± 1.5	0.577
Time and motion	2.0 ± 1.0	2.3 ± 0.5	2.3 ± 1.3	0.779
Instrument	2.6 ± 1.1	2.4 ± 0.8	2.5 ± 1.2	0.945
Use of assistant	2.3 ± 1.1	2.7 ± 0.8	2.4 ± 1.3	0.709
Non-dominant hand	2.5 ± 0.8	3.0 ± 0.8	2.9 ± 1.4	0.656
Flow of operation	2.3 ± 1.1	2.7 ± 0.5	2.7 ± 1.3	0.642
Total	16.7 ± 6.0	18.5 ± 4.0	16.8 ± 7.6	0.774

**Table 2:** Results of OSATS quantity, total number of knots and success rate on the each variable and total score

	Novice (*n* = 10)	Intermediate (*n* = 12)	Advanced (*n* = 11)	*p*-value
Needle directed at a 90-degree angle to tissues	0.14 ± 0.38	0.20 ± 0.42	0.40 ± 0.52	0.305
Needle passed through tissue using a rotation of the wrist/hand motion	0.14 ± 0.38	0.20 0.42	0.30 ± 0.48	0.605
Suture pulled through to leave a ¼“–½” tail	0.14 ± 0.38	0.00 0.00	0.20 ± 0.42	0.329
A single knot throw performed	0.43 ± 0.53	0.20 ± 0.42	0.20 ± 0.42	0.695
Knot tied down squarely	0.43 ± 0.53	0.20 ± 0.42	0.20 ± 0.42	0.695
A second knot throw performed	0.43 ± 0.53	0.20 ± 0.42	0.30 ± 0.48	0.716
A third knot throw performed	0.71 ± 0.49	0.40 ± 0.52	0.70 ± 0.48	0.263
Knots tied snuggly (no air knots)	0.57 ± 0.53	0.40 ± 0.52	0.40 ± 0.52	0.923
suturing with evenly spaced throws	0.57 ± 0.53	0.90 ± 0.32	0.80 ± 0.42	0.408
closure smooth – no bunching of tissues	0.57 ± 0.53	0.80 ± 0.42	0.80 ± 0.42	0.695
Total score	4.14 ± 3.62	3.50 ± 3.14	4.30 ± 2.41	0.475
Total No. of knots	1.4 ± 1.6	0.9 ± 0.6	1.3 ± 1.5	0.727

## Discussion

There were several main findings from this study. First, there was no significant difference among the groups in LESS proficiency after training. Experienced surgeons did not perform significantly better than less experienced surgeons with regard to their OSATS scores. Previous laparoscopic experiences did not change the outcomes. After a brief training course, surgeons who were novices in conventional laparoscopic surgery reached comparable scores to those of experienced laparoscopic surgeons. The educational program used in this study effectively trains surgeons of varying experience in LESS skills.

One might expect LESS proficiency to require experience in minimal invasive surgery, including laparoscopic surgery. Therefore, it is reasonable to hypothesize that experience in laparoscopy surgery would shorten the LESS learning curve. However, our results are to the contrary. These results may be explained by the fact that suturing was selected as the main task. Distinctions in the suturing method between conventional laparoscopy and LESS may have reduced the score gap. The standard suturing and tying methods in conventional laparoscopy are triangular instrumental composition and the “smiley-face” knot technique [11]. However, during LESS, a single incision limits the range of motion and angle of the laparoscopic instruments and causes instrument crowding. These limitations during LESS suturing make triangular instrumental composition difficult. Therefore, experienced laparoscopic surgeons who are familiar with wide triangular compositions during conventional laparoscopy had trouble adjusting to the more limited triangulation in LESS suturing. The instruments were extremely limited during intracorporeal needling and knotting. Crossing manipulation of articulating instruments requires another learning curve that needs to be overcome [5]. These factors had a big impact on all the course of procedure, and which could make no difference among groups. There was no significant difference in total number of knots between the groups. The lower the OSATS quantity score, the better the procedure. Paradoxically, the total OSATS quantity score was highest in the AS group (2). The intrinsic LESS attributes, such as narrow range of motion and crossing manipulation, are very different from those of conventional laparoscopy. Therefore, these skills were novel for all LESS novices, and made the learning curve steep. Although experienced laparoscopic surgeons may be proficient in skills such as depth perception in the operating field, they still had to learn new skills for LESS. Learning the intrinsic technical difficulties of LESS seems to transcend the benefits of having experience in minimally invasive surgery.

LESS surgery may at first seem to be more technically challenging than conventional laparoscopic surgery. However, there are effective educational tools to train surgeons in LESS. Fransen et al. [12] found that short-term box training significantly improved novice trainees’ basic skills in LESS. Sodergren et al. [8] showed that prior training in conventional laparoscopy did not influence learning or proficiency in LESS for novice surgeons. These previous reports agree with our findings that prior surgical experience in laparoscopic surgery does not make it easier to obtain LESS proficiency.

A distinctive feature of this study is that participants were evaluated under biological conditions using a porcine model, which is most similar to the intracorporeal procedure. In contrast, other studies used the dry box trainer to evaluate participants. Under the dry box condition, it is almost impossible to evaluate tissue handling and task performance with regard to space and depth.

Another distinctive feature of this study is that both quantitative and qualitative analyses were performed. Qualitative analyses allowed us to assess parameters like careful tissue/instrument handling, efficient time/motion, and operative flow. These parameters cannot be evaluated through quantitative analysis alone.

A potential limitation of this study was the level of difficulty of the task itself. Cystorrhaphy demands both basic and relatively advanced surgical skills with high dexterity. The LESS cystorrhaphy may have been so difficult that participants in the AS group could not even complete the task. The reason why we selected cystorrhaphy is that it was necessary to increase the task’s difficulty based upon prior findings in the literature. Researchers who conducted previous studies [7] indicated that the basic tasks that were used may not have provided a sufficient challenge to experienced laparoscopic surgeons [7, 13]. If this is true, it limits a study’s assessment function. Two different studies [7, 13] that were conducted using similar tasks produced contrary results. Increasing the complexity of the task should have led to variation between performances. Therefore, we selected cystorrhaphy as the main task. Analyses included those of suturing, needle handling, knotting and the total number of knots. Quantitative analysis was used to assess the whole step, and basic skills including tissue and instrument handling, motion, and operative flow.

Similar results have been observed between LESS and robotic surgery with regard to prior experience in conventional laparoscopic surgery. Regardless of the differences between these types of surgery, experience in conventional laparoscopic surgery does not appear to affect the learning curve in robotic surgery [14, 15]. These results suggest that conventional laparoscopy does not have to be a transitional step to learning another type of minimally invasive surgery.

## Conclusions

Our results suggest that surgical skills acquired in conventional laparoscopy could not appear to transfer directly to LESS. In addition, a surgeon who is a novice in conventional laparoscopic surgery can acquire comparable proficiency in LESS via training compared to more experienced laparoscopic surgeons.

## Abbreviations

LESS: Laparoendoscopic single-site surgery
SATS: Objective structured assessment of technical skills
